# Validated LC‐MS/MS Method for Quantifying the Antiparasitic Nitroimidazole DNDI‐0690 in Preclinical Target Site PK/PD Studies

**DOI:** 10.1002/bmc.70158

**Published:** 2025-06-26

**Authors:** Wietse M. Schouten, Katrien van Bocxlaer, Hilde Rosing, Alwin D. R. Huitema, Jos H. Beijnen, Jadel M. Kratz, Charles E. Mowbray, Thomas P. C. Dorlo

**Affiliations:** ^1^ Department of Pharmacy and Pharmacology Antoni van Leeuwenhoek/The Netherlands Cancer Institute Amsterdam the Netherlands; ^2^ Skin Research Centre, Hull York Medical School, York Biomedical Research Institute University of York York UK; ^3^ Department of Pharmacology Princess Máxima Center for Pediatric Oncology Utrecht the Netherlands; ^4^ Department of Clinical Pharmacy, University Medical Center Utrecht Utrecht University Utrecht the Netherlands; ^5^ Utrecht Institute of Pharmaceutical Sciences Utrecht University Utrecht the Netherlands; ^6^ Drugs for Neglected Diseases Initiative Latin America Rio de Janeiro Brazil; ^7^ Drugs for Neglected Diseases Initiative Geneva Switzerland; ^8^ Department of Pharmacy Uppsala University Uppsala Sweden

**Keywords:** DNDI‐0690, leishmaniasis, nitroimidazole, target site pharmacokinetics, UHPLC‐MS/MS

## Abstract

Understanding the target site pharmacokinetics (PK) of the nitroimidazole analog DNDI‐0690, a potential drug for the neglected parasitic disease leishmaniasis, is important due to the diversity of infected tissue sites and potential drug penetration variability. An ultrahigh‐performance liquid chromatography–tandem mass spectrometry (UHPLC‐MS/MS) method was developed and validated for quantifying DNDI‐0690 in murine biomatrices (plasma, liver, spleen, skin, and skin microdialysate). The method used three protein precipitation sample preparation procedures, tailored for different biomatrices, utilizing a surrogate biomatrix approach. Murine tissues were enzymatically homogenized with a Collagenase A mixture. Chromatographic detection was performed on a C18 column using gradient elution, coupled to a QTRAP6500 quadrupole MS, operating in positive ionization mode. The method demonstrated accurate and precise quantification of all murine biomatrices on the surrogate biomatrix calibration standards, with a high and reproducible total recovery ranging from 75.9% to 94.2% (CV% ≤ 2.5%). Matrix interferences were mitigated with a deuterated internal standard. Stability experiments demonstrated that DNDI‐0690 remained stable in all biomatrices under various conditions. This validated UHPLC‐MS/MS method was successfully used to quantify DNDI‐0690 in a target site murine infection model, demonstrating its suitability for future target site PK studies involving DNDI‐0690.

## 1 Introduction

Leishmaniasis is a neglected parasitic disease, associated with poverty and malnutrition. More than 250,000 new cases were reported in 2022, a number likely severely underreported with true incidence estimates ranging between 700,000 and 1 million cases annually (WHO 2023a, 2023b). Infection by the *Leishmania* (*L*.) parasite can cause various clinical manifestations, ranging from potentially fatal visceral leishmaniasis (VL) in which spleen, liver, and bone marrow are infected, to cutaneous leishmaniasis (CL), leading to disfiguring skin lesions. The clinical manifestation of the disease largely depends on the specific *Leishmania* species involved. After infection, the unicellular parasites are phagocytosed by human macrophages, where the promastigotes transform into the amastigote stage (Borghi et al. 2017; Chang and Dwyer 1976; McGwire and Satoskar 2014). Current options for leishmaniasis treatments are suboptimal, expensive, often ineffective, and prone to resistance or cause severe side effects (Croft and Olliaro 2011; Mowbray 2018). Better, more accessible, and less expensive treatments are needed to combat leishmaniasis, particularly in the world's poorest regions where this parasitic disease is endemic.

Nitroimidazole analogs are a class of small molecules used to treat bacterial and parasitic infections (Löfmark et al. 2010; Stover et al. 2000; Torreele et al. 2010). The antileishmanial activity of one of these, the new chemical entity (NCE) DNDI‐0690, has been investigated against a wide range of *Leishmania* strains in vitro (Thompson et al. 2018). With its broad‐spectrum activity and efficacy by oral route, DNDI‐0690 would be an ideal candidate for treating leishmaniasis caused by geographically diverse parasite species in low‐ and middle‐income countries (van Bocxlaer et al. 2019; Wijnant et al. 2019). The effectiveness of DNDI‐0690 has been demonstrated in a VL‐infected hamster model (Van den Kerkhof et al. 2018) and CL‐infected murine models (van Bocxlaer et al. 2021; Wijnant et al. 2019). In addition, DNDI‐0690 has successfully undergone evaluation in a Phase I single ascending dose clinical trial in healthy volunteers (NCT03929016).

To gain further insight into the human dosing regimen of DNDI‐0690 in different clinical presentations of leishmaniasis, additional data regarding preclinical pharmacokinetics (PK) and pharmacodynamics (PD) of DNDI‐0690 are needed. The sites of infection (spleen and liver for VL and skin for CL) are important, as they are a determining factor for adequate dosing, local drug exposure, and thus treatment efficacy. Therefore, it is essential to conduct target site PK research in the early phases of drug development, to identify exposure–response relationships more accurately for the various presentations of leishmaniasis. To assess the preclinical target site PK of DNDI‐0690, bioanalytical methods are needed that can accurately quantify DNDI‐0690 in murine plasma and tissues infected with *Leishmania* parasites. In addition, microdialysis can assist in determining the free drug concentration at the target site and will further help to interpret these target site PK results. Two methods for quantifying DNDI‐0690 in biological matrices have been previously used (van Bocxlaer et al. 2021; Wijnant et al. 2019). However, these methods have not been validated and suffer from inadequate method development, resulting in low tissue analyte recovery and poor sensitivity, which impedes the quantification of drug concentration in the tissues. A more sensitive and properly validated method is therefore required to gain a deeper understanding of the target site PK/PD relationship for DNDI‐0690 in VL‐ and CL‐infected preclinical models.

In this study, a novel ultra‐high‐performance liquid chromatography–tandem mass spectrometry (UHPLC‐MS/MS) method was developed and validated to quantify DNDI‐0690 in different murine biomatrices (plasma, skin, liver, and spleen) and in microdialysis fluid. To address the challenge of limited access to blank murine biomatrices and microdialysis fluid, a surrogate biomatrix approach was employed, which involved preparing calibration standards and quality control (QC) samples in a substitute blank matrix instead of the authentic matrix (Ho and Gao 2015). For this approach, three different surrogate matrices were used, each with its own specific sample preparation method. The developed bioanalytical method was successfully applied to a preclinical target site PK/PD study in a murine CL infection model. The enhanced sensitivity of the method, favorable tissue analyte recovery, and comprehensive validation across multiple biomatrices enabled this method to provide valuable preclinical PK data on DNDI‐0690 for its potential translation to clinical studies for leishmaniasis.

## 2 Materials and Methods

### 2.1 Chemicals and Biomatrices

**FIGURE 1:**
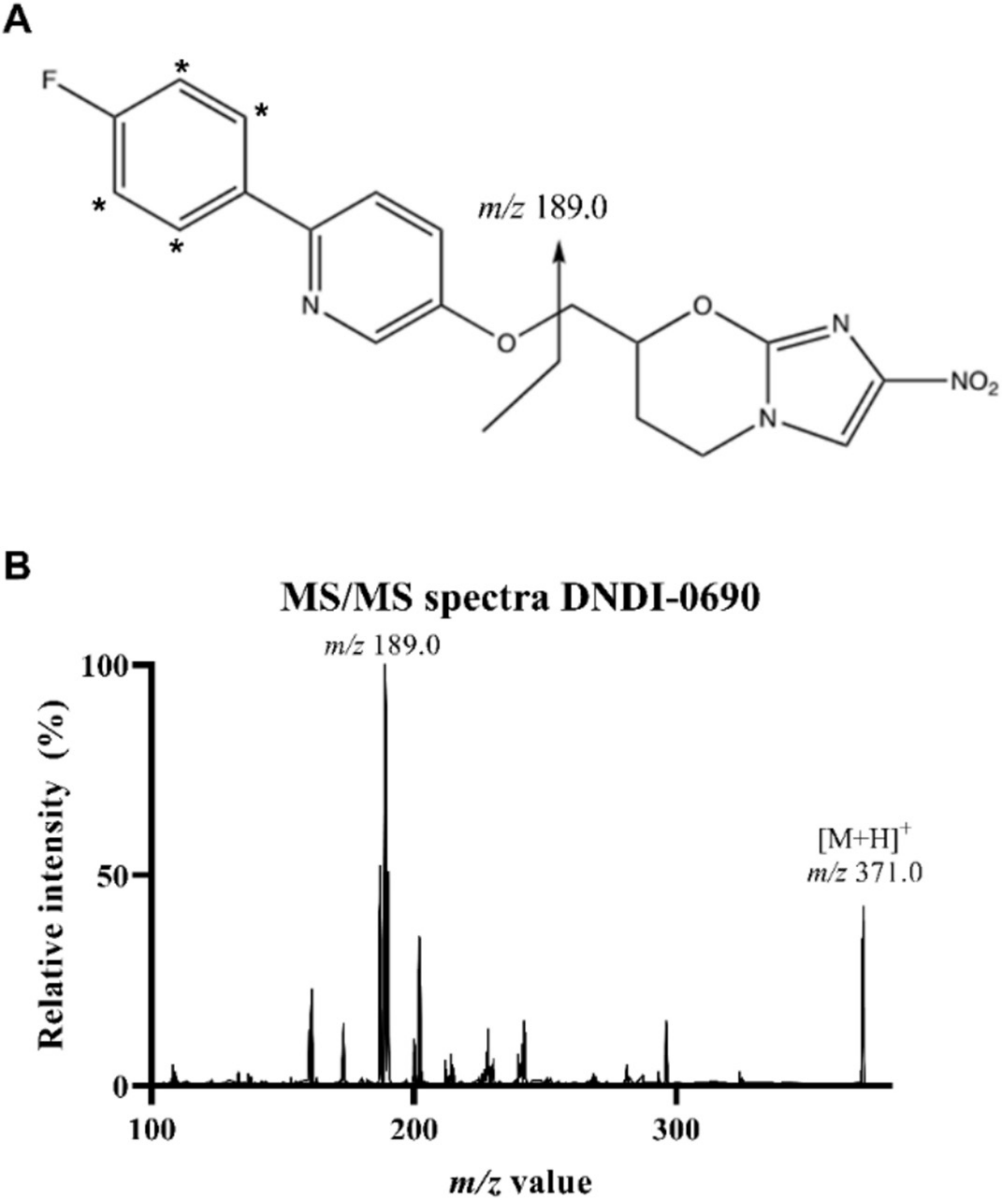
(A) The chemical structure of DNDI‐0690. The arrow indicates the location of the fragmentation of the product ion of *m/z* 189.0, which was used for quantification of DNDI‐0690. The asterisks (*) indicate the location of the deuterated hydrogen atoms of the internal standard [d_4_]‐DNDI‐0690, leading to a product ion of *m/z* 193.0. (B) MS/MS spectrum of the parent ion of DNDI‐0690 (*m/z* 371.0) highlighting the selected production ion at *m/z* 189.0.

ULC/MS grade acetonitrile, methanol, formic acid, and water were all purchased from Biosolve (Valkenswaard, the Netherlands). DNDI‐0690 (Figure 1A) and the stable isotopically labeled internal standard (IS) [d_4_]‐DNDI‐0690 (isotopic purity of 93.3%, containing 1.1% [d_0_]‐DNDI‐0690 and 5.6% [d_3_]‐DNDI‐0690) were provided by the Drugs for Neglected Disease initiative (Geneva, Switzerland). Bovine serum albumin (BSA) Fraction V and Collagenase A were both obtained from Roche (Woerden, the Netherlands). Ringer's solution tablets originated from VWR (Amsterdam, the Netherlands). Blank human K_2_EDTA plasma was obtained from BioIVT (Westbury, NY, United States). Calcium chloride dihydrate and TRIS base were purchased from Sigma Aldrich (Zwijndrecht, the Netherlands). The Enzymatic Collagenase A digestion buffer, prepared as described previously (Roseboom et al. 2022a, 2022b, 2022c), consisted of 5 mg/mL Collagenase A in a 2% BSA in 5‐mM calcium chloride–25‐mM TRIS‐buffer (pH 7.5) and was used for tissue homogenization. Blank microdialysate consisted of a mixture of 2.5% BSA in Ringer's solution—acetonitrile (6:1, *v/v*). Blank murine tissues (skin, liver, and spleen) and murine K_2_EDTA plasma were obtained from the Biological Services Facility at the University of York (York, United Kingdom).

### 2.2 Stock and Working Solutions

DNDI‐0690 and IS stock solutions were prepared in dimethyl sulfoxide (DMSO) by dissolving the compounds in appropriate volumes to obtain a concentration of 1.00 mg/mL. Separate stock solutions were used to prepare the working solutions for calibration standards and QC samples. Working solutions for both DNDI‐0690 and the IS were prepared in methanol. All stock and working solutions were stored at −20°C.

### 2.3 Calibration Standards and QC Samples

Calibration standards (eight concentration levels) and QC samples (QC–lower limit of quantification [LLOQ], QC‐LOW, QC‐MID, and QC‐HIGH) were prepared by diluting the working solutions 20‐fold in their surrogate biomatrices (see Table S2 for calibration standard concentrations and Table 1 for QC concentration levels). The following surrogate biomatrices were used: blank human K_2_EDTA plasma as a substitute matrix for murine plasma, blank enzymatic digestion buffer as a substitute matrix for the murine tissue homogenates, and blank microdialysate as a substitute matrix for the murine skin microdialysate. Spiked human K_2_EDTA plasma samples were kept at −20°C, whereas the spiked enzymatic digestion buffer and microdialysate were stored at −70°C.

**TABLE 1:** Method performance data for DNDI‐0690 from spiked QC samples at LLOQ, LOW, MID, HIGH, and dilution integrity (DIL) concentration levels in blank human K2EDTA plasma, enzymatic digestion buffer, and microdialysate. Accuracy (bias %) and precision (CV%) were assessed at four concentration levels, with quintuplicate measurements in three consecutive runs (*n* = 15). DIL samples were diluted 20 times with the appropriate blank biomatrix, with intrarun accuracy and precision evaluated in a single run.

Matrix	Nom. conc. (ng/mL)	Mean measured conc. (ng/mL)	Intrarun (*n* = 5, in three runs)		Interrun (*n* = 15)	
			Accuracy (bias %)	Precision (CV%)	Accuracy (bias %)	Precision (CV%)
Human K_2_EDTA plasma	2.50	2.55	−3.5 to 12.9	≤ 6.8	2.1	8.9
	7.50	7.71	−2.3 to 12.3	≤ 4.7	2.8	7.9
	75.0	79.3	4.4–8.2	≤ 4.5	5.8	1.4
	750	782	2.5–6.4	≤ 5.8	4.2	0.7
	1.00 × 10^4^	1.03 × 10^4^	2.7	≤ 4.9	—	—
Enzymatic digestion buffer	1.00	0.993	−3.2 to 2.7	≤ 5.7	−0.7	2.6
	3.00	3.22	6.3–9.4	≤ 3.6	7.4	0.8
	40.0	41.8	2.2–6.0	≤ 2.3	4.4	1.8
	375	380	−1.2 to 2.9	≤ 3.4	1.2	1.8
	2.00 × 10^3^	1.96 × 10^3^	−1.9	≤ 1.7	—	—
Microdialysate	0.500	0.502	−2.0 to 2.2	≤ 5.9	0.5	0.5
	1.50	1.47	−4.0 to −0.7	≤ 5.0	−2.0	1.0
	12.5	12.6	0.6–1.8	≤ 3.7	1.1	—^a^
	75.0	74.6	−1.7 to −0.1	≤ 4.1	−0.6	—^a^
	500	504	0.8	≤ 5.4	—	—

Abbreviations: CV%, coefficient of variation; DIL, dilution integrity; LLOQ, lower limit of quantification; nom. conc., nominal concentration.

^a^ No additional variation was found between performing the method on different days (mean square between groups is less then mean square within groups).

### 2.4 Sample Preparation

#### 2.4.1 Murine Plasma

To prepare murine plasma samples, 10‐μL aliquots of calibration standards, QC samples (both in blank human K_2_EDTA plasma), and study samples (in murine plasma) were used. Ninety‐microliter precipitation mixture consisting of 1% (*v/v*) formic acid in water‐acetonitrile (1:8, *v/v*) containing the IS (at a concentration of 1.00 ng/mL) was added to these plasma samples. After briefly vortexing, the samples were centrifuged at 18,600*g* for 10 min, and 75 μL of supernatant was then transferred to an autosampler vial with insert.

#### 2.4.2 Murine Tissues

Murine tissue samples were first weighed using a micro balance. Enzymatic digestion buffer was then added in a 20:1 (*v/w*) enzymatic digestion buffer‐to‐organ weight ratio. The calibration standards and QC samples (both in blank enzymatic digestion buffer) were aliquoted in 50‐μL portions. To all samples, IS (40 ng/mL in the enzymatic digestion buffer) was added in a 1:5 (*v*/*v*) IS‐to‐enzymatic digestion buffer ratio. The samples, including calibration standards, QC samples, and murine tissue samples, were then subjected to a minimum incubation period of 16 h at 37°C, with agitation at 1200 rpm using a Thermomixer (VWR, Amsterdam, the Netherlands) for homogenization. After the incubation period, 60 μL of the homogenized murine tissue sample containing the IS was transferred to a clean tube. The remaining tissue homogenates were preserved at −70°C for potential reanalysis. Following this, 200 μL of an acetonitrile‐methanol (1:1, *v*/*v*) mixture was added to each sample, followed by shaking at 900 rpm for 10 min in a Thermomixer to induce protein precipitation. The samples were then centrifuged for 5 min at 21,000*g*, and 150 μL of the resulting supernatant was transferred to autosampler vials with inserts.

#### 2.4.3 Murine Skin Microdialysate

To prepare the microdialysate samples, 25‐μL aliquots of calibration standards, QC samples (both in blank microdialysate), and study samples (microdialysate collected in murine skin) were used. To all samples, 10 μL of IS (20 ng/mL in methanol) and 65 μL of an acetonitrile‐methanol (1:1, *v/v*) mixture were added. These samples were then gently mixed for 10 min at 900 rpm in the Thermomixer, followed by centrifugation for 5 min at 15,000*g*. Subsequently, 60 μL of the resulting supernatant was transferred to an autosampler vial with insert.

### 2.5 Instrumentation and Chromatographic Conditions

Chromatographic separation and detection were performed using an Agilent 1290 Infinity II system (Agilent Technologies, Santa Clara, CA, United States) coupled to a QTRAP6500 MS (Sciex, Framingham, MA, United States), equipped with a turbo ionspray (Sciex). A gradient elution was applied with 0.1% (*v*/*v*) formic acid in water (Mobile Phase A) and 0.1% (*v*/*v*) formic acid in acetonitrile (Mobile Phase B): from 0–2.5 min, a linear increase from 30% B to 60% B; from 2.5–2.6 min, a linear increase from 60% B to 95% B; isocratic at 95% B for 0.4 min; and then back to 30% B from 3.0 to 3.1 min and conditioning for 0.9 min. The total run time was 4 min with a flowrate set at 0.40 mL/min. An Acquity UPLC BEH C18 column (50 × 2.1 mm, particle size of 1.7 μm) (Waters, Milford, MA, United States) was kept at 40°C, and 2 μL of sample was injected into the system. The MS was operated in positive ionization mode with the parameter settings presented in Table S1. The [M + H]^+^ was selected using multiple reaction monitoring (MRM) for the analyte and the IS, with the following *m/z* transitions: 371.0 → 189.0 (for DNDI‐0690) and 375.0 → 193.0 (for the IS). Instrument operation, data acquisition, and processing were performed using Analyst Software, Version 1.7.2 (Sciex).

### 2.6 Validation Procedure

Full validation of the bioanalytical method to assess DNDI‐0690 concentration in each surrogate biomatrix (human K_2_EDTA, enzymatic digestion buffer, and microdialysate) was carried out in line with the International Council for Harmonisation of Technical Requirements for Pharmaceuticals for Human Use (ICH) guideline M10 on bioanalytical method validation (EMA 2022).

Murine biomatrices (plasma, skin, liver, and spleen) are rare and difficult to acquire. Consequently, only a limited number of validation experiments were conducted for these murine biomatrices, whereas the full validation was performed on the surrogate biomatrices (human K_2_EDTA, enzymatic digestion buffer, and microdialysate), which were used for the preparation of the calibration standards and QC samples. The validation experiments for the murine biomatrices included assessments of accuracy and precision (in a single run), dilution integrity (dilution in the appropriate surrogate biomatrix), endogenous interferences, and stability under different conditions. This was done to ensure that the surrogate biomatrix approach could be employed for the quantification of DNDI‐0690 in these murine biomatrices, without compromising the accuracy and precision of the method developed. Blank murine skin microdialysate could not be obtained due to the invasiveness and complexity of the microdialysis procedure; therefore, no partial validation was conducted for murine skin microdialysate. Instead, full validation was performed using blank microdialysate.

#### 2.6.1 Calibration Curve (Linearity, Range, and LLOQ)

The linearity of the method was determined by preparing eight calibration levels in the surrogate biomatrices (human K_2_EDTA, enzymatic digestion buffer, and microdialysate) and measuring these in duplicate in three separate runs (described in Section 2.3). Linear regression was chosen as the calibration model, using a 1/*x* ^2^ weighting, where *x* represents the analyte concentration. The back‐calculated concentrations of each set of calibration standards should not exceed ± 15% of the nominal concentrations (± 20% for the LLOQ). Furthermore, at least 75% of these calibration standards, and at least one of these duplicate standards, should meet this requirement.

The LLOQ of each surrogate biomatrix was independently determined by calculating the signal‐to‐noise (S/N) ratio. This was done by comparing the noise level of a blank sample with the peak height of an LLOQ sample. The S/N ratio was determined in three separate runs; this ratio should not be lower than 5.

#### 2.6.2 Carryover

Carryover was determined by injection of two double blanks after the upper limit of quantification (ULOQ) calibration standard, comparing the peak area of DNDI‐0690 in the first double blank with the peak area of the LLOQ, which should not exceed 20%. For the IS, this should not exceed 5% of the peak area of the IS in the LLOQ sample.

#### 2.6.3 Accuracy and Precision

The performance of the method was evaluated by analyzing QC samples (QC‐LLOQ, QC‐LOW, QC‐MID, and QC‐HIGH), in quintuplicate in three separate runs. The accuracy and precision of the different sample preparation procedures were assessed in each surrogate biomatrix. The intrarun bias was defined as the deviation of the mean measured concentration in each run from the nominal concentration. The interrun bias was calculated using the mean measured concentration across three different runs, relative to the nominal concentration. Intrarun precision was expressed as the coefficient of variation (CV%), whereas interrun precision was assessed using a one‐way analysis of variance (ANOVA) test. The accuracy and precision values for all three surrogate biomatrices at each concentration level should be ± 15% for accuracy and ≤ 15% for precision, with the exception for LLOQ‐level, which should be ± 20% for accuracy and ≤ 20% for precision.

Due to the limited availability of blank murine plasma and tissues, only intrarun accuracy and precision were determined for these biomatrices. The QC samples (prepared in blank murine K_2_EDTA plasma and tissue homogenates) were quantified in quintuplicate per QC‐level in a single run, using calibration standards prepared in surrogate biomatrices (see Section 2.4). The same requirements used for the surrogate biomatrices were applied to the accuracy and precision evaluations in murine plasma and tissues. This approach was used to demonstrate whether the use of the surrogate matrices for the quantification of DNDI‐0690 in murine biomatrices was justified.

#### 2.6.4 Dilution Integrity

Dilution integrity was determined for the surrogate biomatrices and the murine plasma and tissues. This was evaluated by spiking each biomatrix above the ULOQ. All biomatrices, except the enzymatic digestion buffer and murine tissue homogenates, were diluted 20‐fold with blank surrogate matrix and processed as described in Section 2.4. To mimic dilution integrity for murine tissue study samples, the enzymatic digestion buffer and murine tissue homogenates were first processed as described in Section 2.4 and afterwards diluted 20‐fold with a processed blank sample (containing IS). The bias (%) of the diluted samples compared to the nominal concentration and the precision (CV%) should be ± 15% and ≤ 15%, respectively.

#### 2.6.5 Selectivity and Specificity

To investigate cross‐analyte/IS interference, the peak areas of DNDI‐0690 and the IS in a sample spiked with DNDI‐0690 at the ULOQ level and processed without the IS were compared to the peak areas in a processed blank sample containing only the IS. The peak area of the interference of the IS in the DNDI‐0690 transition should be ≤ 20% of the peak area observed in the QC‐LLOQ sample. Vice versa, the peak area of the interference of DNDI‐0690 in the IS should be ≤ 5% of the IS peak area observed in the QC‐LLOQ sample. This was performed in all surrogate biomatrices.

The selectivity of the method was assessed in all biomatrices, focusing on the endogenous interferences present in the DNDI‐0690 transition induced by the different biomatrices. This was assessed on blank biomatrix and spiked biomatrix at QC‐LLOQ level. For human K_2_EDTA plasma, six different batches were tested. Due to the unavailability of different batches of blank enzymatic digestion buffer, microdialysate, murine plasma, and tissues, only one batch per biomatrix was used to determine the endogenous interferences determination. Endogenous interferences were assessed by comparing the coeluting peaks in the double blanks at the retention time of DNDI‐0690 and the IS with the LLOQ peak area. The peaks in the double blanks should not exceed 20% of the analyte peak area and 5% of the IS peak area of the LLOQ sample. In addition, the bias of the spiked LLOQ samples in each batch of biomatrix should fall within ± 20% of the nominal concentration.

#### 2.6.6 Matrix Effect and Recovery

The matrix effect and recovery were assessed in all surrogate biomatrices, measured at QC‐LOW and QC‐HIGH levels. Six different batches were used for human plasma, whereas only one batch was used for enzymatic digestion buffer and microdialysate (*n* = 6 samples per QC level). The matrix factor (MF) was determined by comparing the peak area of DNDI‐0690 and the IS in the matrix present sample (MPS, sample spiked post‐sample preparation of blank surrogate biomatrix) with the peak area in the matrix absent sample (MAS, sample spiked post‐sample preparation without blank biomatrix). Calculation of the MF involved dividing the peak area of a single MPS by the mean peak area of the MAS. To obtain the IS‐normalized MF, the MF of DNDI‐0690 was divided by the IS‐MF.

Recovery of the sample preparation was determined by comparing the peak area of DNDI‐0690 in a processed sample with that of the MPS. The total recovery was calculated by taking the ratio of the peak area of the analyte in a processed sample to that of the MAS. These recoveries were also calculated for the IS and then normalized against the DNDI‐0690 recoveries to obtain IS‐normalized recoveries. Each recovery experiment was performed in triplicate at QC‐LOW and QC‐HIGH levels.

#### 2.6.7 Stability Assessment

The stability of DNDI‐0690 was assessed under different conditions. The condition was stable if the measured mean concentration was within ± 15% of the nominal concentration and exhibited a CV% of ≤ 15%, when tested on a set of freshly prepared calibration standards. Acceptable stability for stock and working solutions was defined as a response within ± 5% of the response of freshly prepared solutions. The stability (*n* = 3 per QC level, per condition) in surrogate biomatrices was evaluated at QC‐LOW and QC‐HIGH levels, whereas the stability in murine plasma and tissue homogenates was tested at QC‐MID level.

The stability of DNDI‐0690 in the surrogate biomatrices, murine plasma and murine tissue homogenates was tested when stored under processing conditions (room temperature, nominally 21°C for 8–24 h), under long‐term storage conditions (at nominally −20°C or −70°C for 9–65 days) and after three freeze (at nominally −20°C or −70°C)/thaw (at room temperature) (F/T) cycles. In addition, the effect of the murine tissue enzymatic digestion workflow on the stability of DNDI‐0690 in murine tissue homogenates was tested at 37°C for at least 16 h in enzymatic digestion buffer in the presence of Collagenase A.

### 2.7 Preclinical Application: Target Site PK Study in Murine CL Infection Model

A target site PK study in an *L. major* murine CL infection model was conducted to determine whether the method was applicable to measuring DNDI‐0690 in murine biomatrices. For this study, female BALB/c mice (6–8 weeks old) were purchased from Charles River Laboratories (Margate, United Kingdom). Mice were housed in groups of five in ventilated cages in a controlled environment of 56% relative humidity, 20°C–21°C and a 12:12 light:dark cycle. They were provided with tap water and a standard ad libitum laboratory diet and were left to acclimatize for 5 days prior to the beginning of the study.

All animal work was carried out under project license PP1651724 at the Biological Services Facility (University of York, United Kingdom) and was in accordance with the Animal (Scientific Procedure) Act 1986 under a UK Home Office project license according to the European Directive on the protection of animals used for scientific purposes 2010/63/EU. The study protocol and procedures underwent examination and approval from the Animal Welfare and Ethical Review Board (University of York, United Kingdom).

#### 2.7.1 Target Site Pharmacokinetics

First, stationary phase Ppy RE9H + *L. major* Friedlin (MHOM/IL/81/Friedlin) promastigotes were injected into the shaved rump of BALB/c mice. After the appearance of skin lesions, the nodules were measured until they reached a diameter of 6 mm. The mice were subsequently given the 10‐day oral DNDI‐0690 treatment (50 mg/kg, qd). Blood samples (*n* = 3 per time point) were collected at the following time points postdosing: 0.5, 1.0, 2.0, 4.0, 8.0, 12.0, and 24.0 h on both Day 1 (D1) and Day 10 (D10) of treatment. At each time interval, 50 μL of blood was collected into EDTA K3E‐coated collection tubes (Sarstedt, Nümbrecht, Germany). The samples were kept at RT for 30 min and then centrifuged at 1000×*g* for 15 min. The resulting plasma was transferred into clean tubes and stored at −70°C. The mice were sacrificed at 24.0 h after D10 of treatment, and two skin samples, infected and noninfected skin as well as liver and spleen, were all excised. All samples were directly stored at −70°C before transport. All samples were processed and analyzed as described above (Sections 2.4 and 2.5). Plasma PK parameters (area under the plasma concentration‐time curve from 0 until 24 h [AUC_0–24h_], maximum concentration [C_max_], and the skin accumulation difference) were determined using Prism Software, Version 9.3.0 (GraphPad, Boston, MA, United States). Concentrations of DNDI‐0690 (in ng/mL) measured in infected and noninfected skin tissue homogenates were converted to concentrations of DNDI‐0690 in skin tissue (ng/g). A paired *t* test was performed to assess statistical differences in drug accumulation between infected and noninfected skin from three individual mice.

#### 2.7.2 Target Site Microdialysis

The *Leishmania*‐infected mice were also subjected to dermal microdialysis experiments. Prior to putting MAB 1.2.4. Cu probes (6 kDa‐cutoff cuprophane membrane, Microbiotech/se AB, Sweden) in the dermal layer of infected and noninfected skin with a 22‐gauge needle, mice were first sedated using an urethane:chlorprothixine hydrochloride (CPX) mixture (ip, 1.5 g/kg urethane and 5 mg/kg CPX) and placed on a temperature‐controlled heating pad (VetTech, Cheshire, United Kingdom) maintaining the body temperature of the mice at 32°C ± 2°C. Before collecting the samples, a 30‐min stabilization period of perfusion with 2.5% BSA in Ringer's solution at a flow rate of 2 μL/min with a CMA 402 syringe pump (Biochrom Ltd, United Kingdom) was used to equilibrate the system and allow the skin to recuperate from the probe insertion trauma. Mice were orally administered 50 mg/kg DNDI‐0690 at the start of the skin microdialysis experiment. Skin microdialysate was captured over a 30‐min period, for 6 h in total, using a refrigerated microdialysis fraction collector (MAB 85, Microbiotech/se AB) set at 4°C, yielding a total of 12 microdialysate samples per skin probe. To increase solubility of the compound, 10‐μL acetonitrile was added to each microdialysate sample collected. All the skin microdialysate samples were immediately stored at −20°C during the experiment and transferred to −70°C after the experiment, before being processed and analyzed as described above (Sections 2.4 and 2.5).

## 3 Results and Discussion

### 3.1 Development of the Bioanalytical Method

A reversed phase Acquity BEH C18 UHPLC column (50 × 2.1 mm, particle size of 1.7 μm) was used for chromatography. A symmetrical peak (asymmetry factor of 1.3) eluting after 1.8 min was obtained when a linear gradient from 30% to 60% Mobile Phase B was applied over 2.5 min. The quadrupole MS was operated in the positive ionization mode for detecting and to quantifying DNDI‐0690. The single‐charged [M + H]^+^ of DNDI‐0690 was observed, and the most abundant transition was at *m/z* 371.0 → 189.0. The IS, [d_4_]‐DNDI‐0690, was monitored at *m/z* 375.0 → 193.0. The Q3 spectrum for DNDI‐0690 and the proposed product ion are both depicted in Figure 1A,B. This chromatographic setup allowed quantification of DNDI‐0690 in different biomatrices down to 0.500 ng/mL. During method development, the IS was found to contain a small percentage (~1%) of undeuterated DNDI‐0690, indicating suboptimal isotopic labeling. The isotopic purity was 93.3%, with the remaining 6.7% consisting of undeuterated DNDI‐0690 and [d_3_]‐DNDI‐0690 as minor isotopic impurities. To minimize cross‐analyte interference at the DNDI‐0690 transition, the IS in the final extract was limited to a maximum of 2.00 ng/mL.

We recently published a tissue homogenization workflow using a collagenase A‐based enzymatic digestion buffer for the homogenization of human skin (Roseboom et al. 2022a, 2022b, 2022c). This workflow was successfully used to obtain skin tissue homogenates for the quantification of clinical antileishmanial drugs. We have evaluated and validated the enzymatic homogenization method for murine tissues, which demonstrated improved tissue homogenization and yielded higher absolute analyte extraction compared to commonly used mechanical homogenization (Schouten et al. 2025). Given these superior results, we further assessed the suitability of Collagenase A–based enzymatic digestion for tissue homogenization and extraction of DNDI‐0690 from murine skin study samples. Enzymatic homogenization resulted in a 9.6‐fold higher tissue concentration than mechanical homogenization, indicating that the former extracts the compound more effectively from the skin tissue.

**FIGURE 2:**
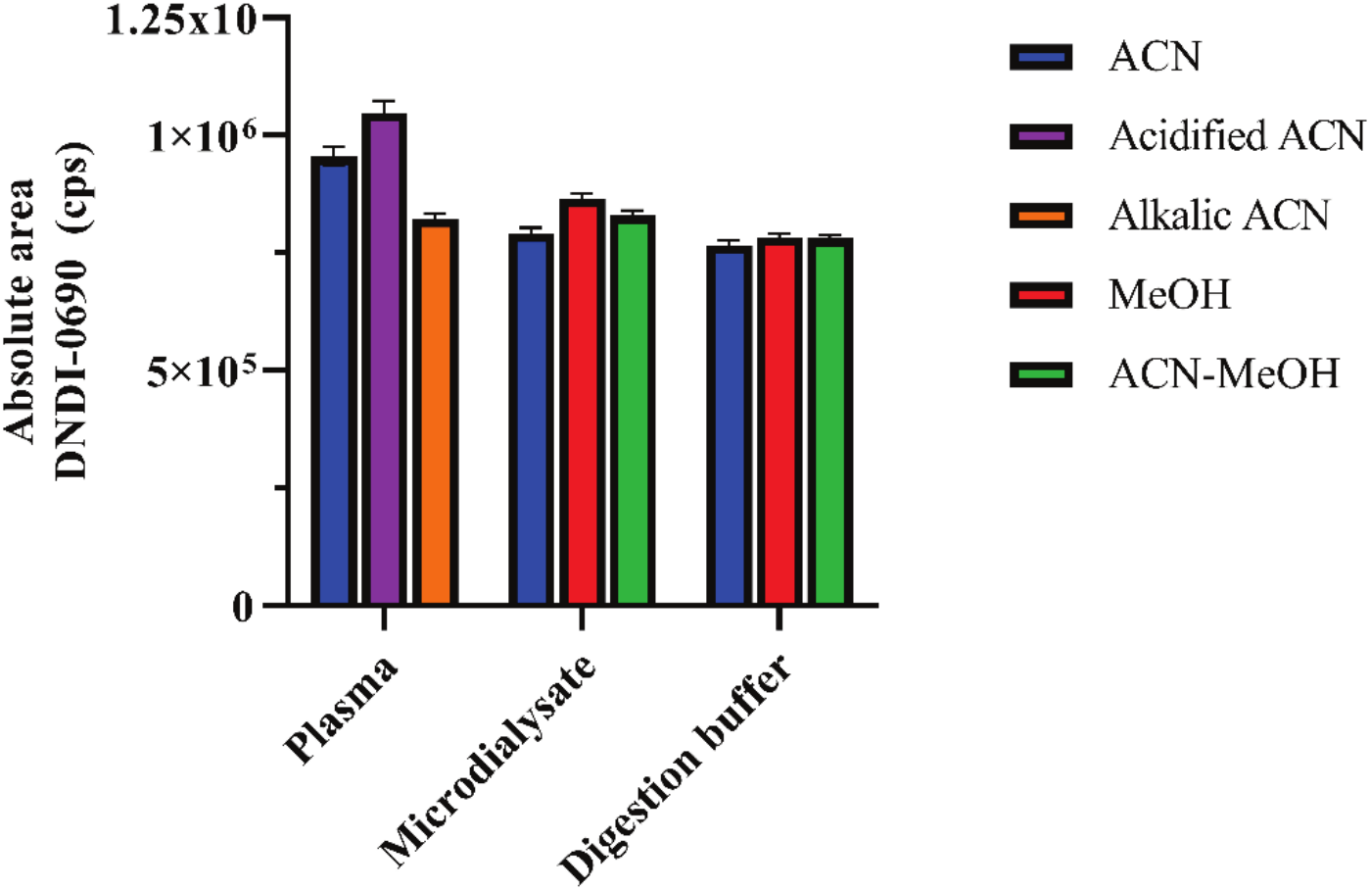
Optimization of protein precipitation for each different biomatrix; human K_2_EDTA plasma (spiked at 75.0 ng/mL), digestion buffer, and microdialysate (spiked at 50.0 ng/mL). Each protein precipitation procedure was tested and analyzed in triplicate. Abbreviations: ACN, acetonitrile; MeOH, methanol.

Various protein precipitation sample preparation methods were empirically investigated for further DNDI‐0690 sample clean‐up (*n* = 3 per condition). In total, three different organic solvent mixtures (acetonitrile, methanol, and acetonitrile‐methanol [1:1, *v*/*v*]) were tested for enzymatic digestion buffer and microdialysate. Because a higher response was needed for plasma due to the low sample volume, different compositions of acetonitrile, under acidic and alkaline conditions, were tested. The precipitation mixture with the highest response and the lowest variability was selected for each biomatrix: acidified acetonitrile for plasma, acetonitrile‐methanol for enzymatic digestion buffer, and methanol for microdialysate (Figure 2).

Surrogate biomatrices were used for the quantification of murine plasma and tissues. During the development phase, preliminary accuracy and precision experiments were conducted to assess the suitability of the use of these surrogate biomatrices. In these experiments, blank murine biomatrices (K_2_EDTA plasma and digested skin, spleen, and liver homogenates) were spiked at all QC levels and quantified on a surrogate calibration curve. All measured QC levels had biases of ± 15% and precisions of ≤ 15% (data not shown), which was deemed acceptable based on the guidelines. However, a decrease (maximum of 15.2%) in the analyte response was observed in all digested murine tissues at all QC levels, suggesting potential matrix effects or reduced recovery. The use of a stable isotopically‐labeled IS effectively addressed this analyte response issue, as demonstrated by the accurate quantification of the QC samples prepared in murine tissue homogenates. The LLOQ of 1.00 ng/mL was still quantifiable in all these biomatrices except microdialysate and had S/N ratios of > 5.

### 3.2 Validation Procedures

#### 3.2.1 Calibration Curve (Linearity, Range, and LLOQ)

**FIGURE 3:**
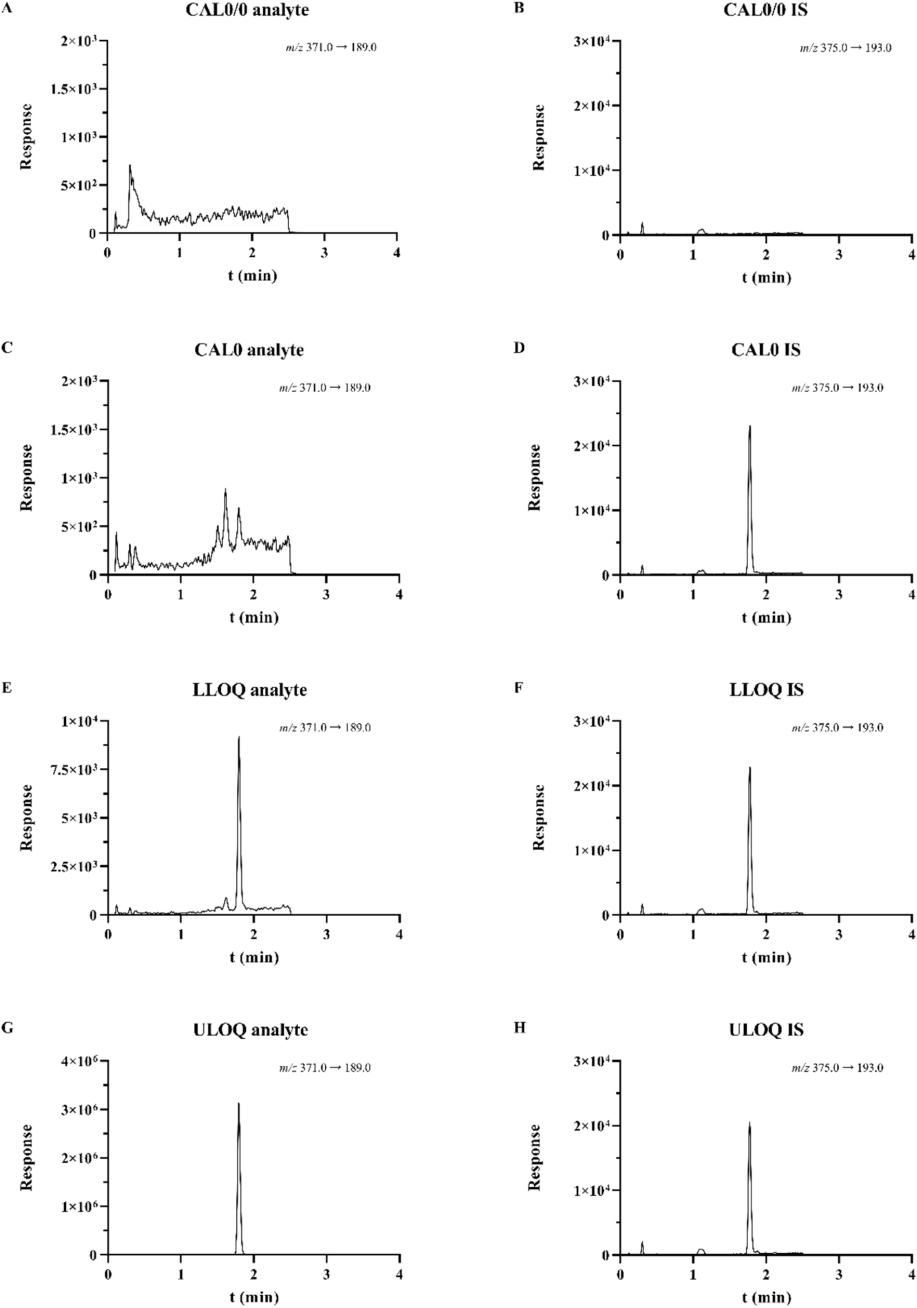
Representative MRM chromatograms of DNDI‐0690 and the IS for the double blank (A, B), the blank (C, D), the lower limit of quantification (LLOQ) of 2.50 ng/mL (E, F), and the upper limit of quantification (ULOQ) of 1000 ng/mL (G, H), at their respective transitions in human K_2_EDTA plasma.

Linearity was observed over a concentration range of 2.5–1000 ng/mL for the plasma matrix, 1.0–500 ng/mL for the tissue matrix, and 0.5–100 ng/mL for the microdialysate matrix. All back‐calculated concentrations of the calibration standards were in accordance with the acceptance criteria (Table S2). For all surrogate biomatrix calibration curves, the correlation coefficient (*R* ^2^) of the linear fit was at least 0.9988, as assessed in three separate analytical runs. All validated methods had an S/N ratio at an LLOQ level of at least 5.0. Representative MRM chromatograms of DNDI‐0690 and the IS retrieved from the plasma sample preparation method are shown in Figure 3.

#### 3.2.2 Carryover

There was no carryover observed in any of the matrices for either DNDI‐0690 or the IS.

#### 3.2.3 Accuracy and Precision

A detailed overview of the accuracy and precision data for each surrogate biomatrix is included in Table 1. The intrarun and interrun accuracy ranged between −4.0% and +12.9%, respectively, and the intrarun and interrun precision was ≤ 8.9 for all surrogate biomatrices at all QC levels. Therefore, all acceptance criteria of ± 15% for bias and ≤ 15% for precision were met.

Due to the lack of murine plasma and tissues, only the intrarun accuracy and precision were determined (Table 2). All murine biomatrices were quantifiable using the surrogate matrix calibration standards with accuracy and precision values within ± 15% and ≤ 15%, respectively. Consequently, murine plasma could be quantified using calibration standards prepared in human K_2_EDTA plasma, and murine tissue homogenates (skin, liver, and spleen) on calibration standards prepared in enzymatic digestion buffer.

**TABLE 2:** Method performance data for the partial validation of DNDI‐0690 obtained from spiked QC samples (LLOQ, LOW, MID, HIGH, and dilution integrity [DIL] concentration levels) in blank murine K_2_EDTA plasma and murine skin, liver, and spleen homogenates. The accuracy (bias %) and the precision (CV%) were analyzed at five concentrations in quintuplicate in a single run.

Matrix	Nom. conc. (ng/mL)	Mean measured conc. (ng/mL)	Accuracy (bias %)	Precision (CV%)
Murine K_2_EDTA plasma	2.50	2.69	7.5	5.5
	7.50	7.65	2.0	2.5
	75.0	78.8	5.1	1.1
	750	770	2.6	1.8
	1.00 × 10^4^ (DIL)	1.06 × 10^4^	5.8	3.9
Murine skin homogenate	1.00	1.15	14.8	1.9
	3.00	3.20	6.8	2.9
	40.0	40.9	2.3	2.0
	375	376	0.3	1.3
	2.00 × 10^3^ (DIL)	1.97 × 10^3^	−1.3	1.1
Murine liver homogenate	1.00	0.946	−5.4	5.7
	3.00	3.13	4.4	1.9
	40.0	41.3	3.2	1.3
	375	375	0.1	0.8
	2.00 × 10^3^ (DIL)	2.00 × 10^3^	0.1	2.1
Murine spleen homogenate	1.00	0.966	−3.4	3.4
	3.00	3.00	0.1	2.8
	40.0	40.3	0.7	3.6
	375	371	−1.1	0.7
	2.00 × 10^3^ (DIL)	1.96 × 10^3^	−2.0	2.1

Abbreviations: CV%, coefficient of variation; DIL, dilution integrity; LLOQ, lower limit of quantification; nom. conc., nominal concentration.

#### 3.2.4 Dilution Integrity

Dilution integrity was evaluated by determining whether samples exceeding the ULOQ could undergo a 20‐fold dilution with blank matrix and still be accurately quantified. The dilution integrity of all biomatrices, including surrogate biomatrices, was assessed, and the results are presented in Tables 1 and 2. All values fell within the acceptance criteria, with the intrarun bias ± 5.8% from the nominal concentration and the intrarun precision ≤ 5.4%. This demonstrated that study samples exceeding the calibration range of the methods could be diluted with biomatrix and still be precisely and accurately quantified.

The bias and precision of the 20‐fold dilutions were found to be ± 2.7% and ≤ 4.9%, ± 1.9% and ≤ 1.7%, and ± 0.8% and ≤ 5.4% for the surrogate biomatrices human K_2_EDTA plasma, enzymatic digestion buffer, and microdialysate, respectively.

Additionally, the dilution integrity of murine plasma and tissue homogenates was also assessed; the results are presented in Table 2. All values fell within the acceptance criteria, with the bias ± 5.8% from the nominal concentration and the precision ≤ 3.9%. This demonstrated that study samples exceeding the calibration range of the methods could be diluted in the appropriate surrogate biomatrix and still be precisely and accurately quantified.

#### 3.2.5 Selectivity and Specificity

During the developmental phase of the methods, interference from the IS was discovered in the DNDI‐0690 transition at the same retention time. This interference was observed in all surrogate biomatrices and was attributed to the IS stock. To address this issue, a reduced concentration of the IS was used in each method. As a result, the peak area of DNDI‐0690 in a processed blank sample did not surpass 10.1% of the peak area of DNDI‐0690 in the LLOQ sample, and there was no interfering peak in the IS transition from DNDI‐0690.

The double blanks did not contain endogenous interferences exceeding the peak area of 20% (for DNDI‐0690) or 5% (for the IS) compared to the LLOQ response. Deviation from the LLOQ concentration was between −14.1% and +14.8% for all tested biomatrices. The results were in accordance with the acceptance criteria.

#### 3.2.6 Matrix Effect and Recovery

The MF of DNDI‐0690 and the IS‐normalized MF were determined at QC‐LOW and QC‐HIGH levels for all surrogate biomatrices (Table 3). The MF of DNDI‐0690 ranged from 0.89 to 1.04 (CV ≤ 4.7%). These results suggest that DNDI‐0690 experienced some ion suppression, in particular from the enzymatic digestion buffer and microdialysate biomatrices. The IS‐normalized MFs at all concentrations measured were between 0.99 and 1.08 (CV ≤ 4.3%), indicating that the addition of IS effectively corrected for the biomatrix‐induced ion suppression.

**TABLE 3:** Matrix factor and recovery (in %) for DNDI‐0690 in each surrogate biomatrix, determined at QC‐LOW and QC‐HIGH levels.

Biomatrix	Nom. conc. (ng/mL)	MF DNDI‐0690 (CV%)	IS‐normalized MF (CV%)	Sample preparation recovery (CV%)	Overall recovery (CV%)	IS‐corrected overall recovery
Human K_2_EDTA plasma	15.0	1.04 (1.7%)	0.99 (4.0%)	75.6% (1.0%)	79.4% (0.9%)	100.6% (3.2%)
	1500	1.01 (0.6%)	1.01 (0.9%)	76.7% (2.1%)	75.9% (0.9%)	101.6% (0.4%)
Enzymatic digestion buffer	3.00	0.91 (1.4%)	1.05 (1.6%)	91.2% (0.6%)	92.4% (2.5%)	103.3% (4.2%)
	375	0.92 (0.5%)	1.04 (1.8%)	102.4% (1.9%)	94.2% (2.1%)	105.8% (0.8%)
Microdialysate fluid	1.50	0.91 (4.7%)	1.08 (4.3%)	97.6% (4.5%)	91.1% (1.8%)	102.4% (0.3%)
	75.0	0.89 (1.8%)	1.03 (1.9%)	99.3% (2.6%)	87.5% (2.1%)	98.4% (1.6%)

Abbreviations: CV%, coefficient of variation; IS, internal standard; MF, matrix factor; nom. conc., nominal concentration.

Recovery of the protein precipitation ranged between 75.6% and 76.7% (CV% ≤ 2.1%) for human K_2_EDTA plasma, 91.2% and 102.4% (CV% ≤ 1.9%) for the enzymatic digestion buffer, and 97.6% and 99.3% (CV% ≤ 4.5%) for the microdialysate, respectively (Table 3). The total overall recovery, which also accounts for matrix effect, was similar for human K_2_EDTA plasma. However, a difference (max 11.8%) was observed for the other surrogate biomatrices. Nevertheless, the recovery values for the IS were similar to those for DNDI‐0690 in the biomatrices tested, resulting in an IS‐corrected total recovery of 99.8%–105.8% for all biomatrices (Table 3). In addition, there was low variability between the recovery values (CV% ≤ 4.2%) for all surrogate biomatrices, indicating the sample preparation for each biomatrix was highly reproducible.

#### 3.2.7 Stability Assessment

The stability of DNDI‐0690 under various conditions is summarized in Table 4. DNDI‐0690 was stable in all biomatrices for three F/T cycles. DNDI‐0690 was stable when stored under processing conditions (at 20°C for 6–24 h) and during long‐term storage (at −20°C or −70°C for 9–65 days) for all tested biomatrices. An additional experiment was conducted to investigate the effect on the stability of DNDI‐0690 in enzymatic digestion buffer and digested tissue homogenates during the enzymatic tissue homogenization workflow (37°C for 16 h, in the presence of Collagenase A) with the addition of IS after incubation. These results indicated that there was a loss of DNDI‐0690 analyte response in the murine spleen homogenate during the incubation period, which led to a bias of −16.5% from the nominal DNDI‐0690 concentration. However, the addition of IS prior to incubation corrected for the loss of DNDI‐0690 during enzymatic homogenization in the murine spleen homogenate (data not shown). DNDI‐0690 was also stable in stock solution (DMSO) and working solution (methanol), when stored at nominally −20°C.

**TABLE 4:** Stability parameters for DNDI‐0690 in biomatrix, stock solution, and working solution expressed as the accuracy (bias %) and precision (CV%) (*n* = 3 samples per concentration level).

Matrix	Condition	Nom. conc. (ng/mL)	Accuracy (bias %)	Precision (CV%)
Human K_2_EDTA plasma	−20°C, 34 days	7.50	10.0	0.7
		750	2.8	0.6
	RT, 24 h	7.50	7.4	3.9
		750	2.0	3.1
	3 F/T	7.50	9.1	3.7
		750	7.7	3.3
Murine K_2_EDTA plasma	−70°C, 65 days	75.0	12.8	8.3
	RT, 24 h	75.0	5.7	1.7
	3 F/T	75.0	5.4	1.8
Enzymatic digestion buffer	−70°C, 16 days	3.00	11.1	1.1
		375	3.9	1.7
	RT, 24 h	3.00	10.9	2.5
		375	2.4	3.1
	3 F/T	3.00	12.7	1.8
		375	3.6	2.2
	37°C, 16 h	3.00	0.7	1.5
		375	1.0	0.8
Murine skin tissue homogenate	−70°C, 9 days	40.0	0.1	1.4
	RT, 6 h	40.0	5.3	4.0
	3 F/T	40.0	2.2	1.2
	37°C, 16 h	40.0	−9.8	5.5
Murine liver homogenate	−70°C, 9 days	40.0	2.7	5.1
	RT, 6 h	40.0	1.7	1.6
	3 F/T	40.0	10.8	3.8
	37°C, 16 h	40.0	−3.1	7.8
Murine spleen homogenate	−70°C, 9 days	40.0	−0.3	1.1
	RT, 6 h	40.0	5.3	2.2
	3 F/T	40.0	0.7	5.4
	37°C, 16 h	40.0	−16.5	2.0
Microdialysate fluid	−70°C, 14 days	1.50	8.2	3.7
		75.0	6.4	0.3
	RT, 24 h	1.50	8.2	3.0
		75.0	7.2	1.0
	3 F/T	1.50	7.8	5.5
		75.0	4.8	3.5
DMSO (stock solution)	−20°C, 175 days	1.00 × 10^6^	−4.5	1.0
Methanol (working solution)	−20°C, 34 days	50.0	2.7	1.4
		2.00 × 10^4^	−0.9	0.5

Abbreviations: CV%, coefficient of variation; F/T, freeze/thaw cycles; nom. conc., nominal concentration; RT, room temperature, at 21°C.

### 3.3 Preclinical Application

**FIGURE 4:**
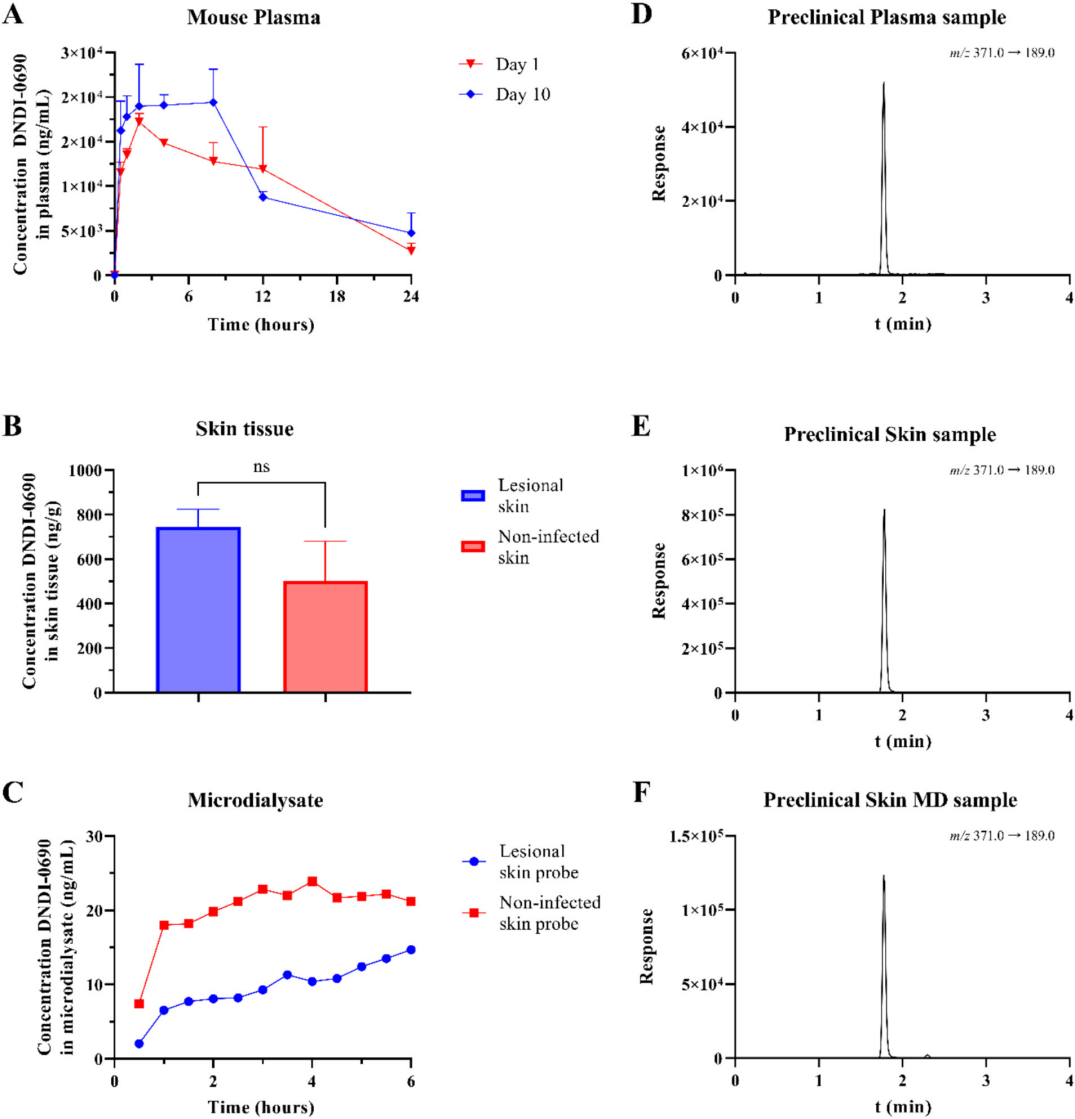
Determination of DNDI‐0690 in different murine biomatrices in a preclinical experiment, where all mice received 50 mg/kg (qd, po) for 10 days. (A) DNDI‐0690 plasma concentration‐time profile of *Leishmania*‐infected mice (*n* = 3) after 1‐ or 10‐day treatment. (B) DNDI‐0690 infected and noninfected skin tissue concentrations at 24 h after a 10‐day treatment (*n* = 3). (C) DNDI‐0690 concentration in microdialysis samples retrieved for the infected and noninfected skin probe after administration of one dose. (E, F) Representative MRM chromatograms for each murine biomatrix measured (murine K_2_EDTA plasma, skin homogenate, and skin microdialysate).

To demonstrate the applicability of the developed method, murine samples from a target site PK study were analyzed. The plasma PK time‐concentration curves for D1 and D10 are depicted in Figure 4A. The total plasma exposure and maximum concentration were higher after D10 and then D1 (AUC_D1,0–24h_ of 249 ± 31 μg·h/mL vs. AUC_D10,0–24h_ of 283 ± 19 μg·h/mL, C_max,D1_ of 17.4 ± 0.8 μg/mL vs. C_max,D10_ of 28.7 ± 4.3 μg/mL). The skin tissue concentrations of DNDI‐0690 for infected and noninfected skin are shown in Figure 4B. Although the difference was not statistically significant (*p* value of 0.0517), there appears to be a trend indicating that the total skin concentration of DNDI‐0690 in infected skin is higher than in noninfected skin.

Murine skin microdialysate samples were collected for 30 min over a period of 6 h after administration of the drug to determine the unbound drug concentration in infected and noninfected skin. The measured unbound drug DNDI‐0690 concentrations are shown in Figure 4C.

None of the preclinical samples, including microdialysis samples collected from the skin where low concentrations were anticipated, showed DNDI‐0690 levels below the LLOQ. This demonstrates that the sample preparation procedures and the UHPLC‐MS/MS method are sensitive enough to meet the requirements for supporting preclinical studies. This demonstrates that the sample preparation procedures and the UHPLC‐MS/MS method are sufficiently sensitive for its intended goal of supporting preclinical studies. Representative MRM chromatograms of the preclinical samples measured are shown in Figure 4D (murine plasma), Figure 4E (murine skin tissue), and Figure 4F (murine skin microdialysate). In addition, the plasma procedure has been validated for the quantification of DNDI‐0690 in human K_2_EDTA, making the developed method suitable for future human plasma PK research on DNDI‐0690.

## 4 Conclusion

An UHPLC‐MS/MS method was validated for measuring the concentration of antiparasitic nitroimidazole DNDI‐0690 in various biomatrices, supporting preclinical research on new treatment options for VL and CL. The method used three different surrogate biomatrices, each requiring specific sample preparation procedures, to analyze DNDI‐0690 in murine K_2_EDTA plasma, tissues (skin, liver, and spleen), and skin microdialysate. Efficient and reproducible tissue homogenization and extraction of DNDI‐0690 from the various murine tissues were achieved using collagenase A‐based enzymatic homogenization, followed by protein precipitation for sample clean‐up. This resulted in a reproducible overall recovery without significant matrix effects. DNDI‐0690 demonstrated stability under several conditions in all investigated biomatrices. The addition of IS prior to incubation effectively corrected for the analyte loss during the homogenization of murine tissues. The method was successfully applied to assess the target site PK of DNDI‐0690 in a preclinical CL‐infected murine model, providing valuable data to support its potential as CL treatment.

## Author Contributions

**Wietse M. Schouten:** conceptualization, methodology, investigation, validation, writing – original draft, writing – review and editing. **Katrien van Bocxlaer:** resources, writing – review and editing. **Hilde Rosing:** conceptualization, methodology, validation, writing – review and editing, supervision. **Alwin D. R. Huitema:** supervision, writing – review and editing. **Jos H. Beijnen:** supervision, writing – review and editing. **Jadel M. Kratz:** resources, writing – review and editing, project administration, funding acquisition. **Charles E. Mowbray:** resources, writing – review and editing, project administration, funding acquisition. **Thomas P. C. Dorlo:** conceptualization, methodology, validation, writing – review and editing, supervision, project administration, funding acquisition.

## Conflicts of Interest

The authors declare no conflicts of interest.

## Supporting information


**Table S1** Above: general mass spectrometric parameters. Below: Analyte specific mass spectrometric parameters for DNDI‐0690 and [d_4_]‐DNDI‐0690.
**Table S2** Calibration standard concentrations, back‐calculated accuracy (Bias, %) and precision (CV, %) of DNDI‐0690 (in ng/mL) for the three different sample preparation methods, analyzed in three consecutive analytical runs.

## Data Availability

The data that support the findings of this study are available from the corresponding author upon reasonable request.

## References

[bmc70158-bib-0001] Borghi, S. , V. Fattori , I. Conchon‐Costa , P. Pinge‐Filho , W. Pavanelli , and W. Verri . 2017. “ *Leishmania* Infection: Painful or Painless?” Parasitology Research 116: 465–475. 10.1007/s00436-016-5340-7. 27933392

[bmc70158-bib-0002] Chang, K.‐P. , and D. M. Dwyer . 1976. “Multiplication of a Human Parasite (*Leishmania donovani*) in Phagolysosomes of Hamster Macrophages In Vitro.” Science 193, no. 4254: 678–680. 10.1126/science.948742. 948742

[bmc70158-bib-0003] Croft, S. L. , and P. Olliaro . 2011. “Leishmaniasis Chemotherapy—Challenges and Opportunities.” Clinical Microbiology and Infection 17, no. 10: 1478–1483. 10.1111/j.1469-0691.2011.03630.x. 21933306

[bmc70158-bib-0004] EMA . 2022. “ICH Guideline M10 on Bioanalytical Method Validation—Step 5.” Accessed 09‐02‐2025, from https://www.ema.europa.eu/en/documents/scientific‐guideline/ich‐guideline‐m10‐bioanalytical‐method‐validation‐step‐5_en.pdf.

[bmc70158-bib-0005] Ho, S. , and H. Gao . 2015. “Surrogate Matrix: Opportunities and Challenges for Tissue Sample Analysis.” Bioanalysis 7, no. 18: 2419–2433. 10.4155/bio.15.161. 26395421

[bmc70158-bib-0006] Löfmark, S. , C. Edlund , and C. E. Nord . 2010. “Metronidazole Is Still the Drug of Choice for Treatment of Anaerobic Infections.” Clinical Infectious Diseases 50: S16–S23. 10.1086/647939. 20067388

[bmc70158-bib-0007] McGwire, B. S. , and A. R. Satoskar . 2014. “Leishmaniasis: Clinical Syndromes and Treatment.” QJM: An International Journal of Medicine 107, no. 1: 7–14. 10.1093/qjmed/hct116. 23744570 PMC3869292

[bmc70158-bib-0008] Mowbray, C. 2018. “Chapter 2: Anti‐Leishmanial Drug Discovery: Past, Present and Future Perspectives.” In Drug Discovery for Leishmaniasis, edited by L. Rivas and C. Gil , 24–36. Royal Society of Chemistry. 10.1039/9781788010177-00024.

[bmc70158-bib-0009] Roseboom, I. C. , B. Thijssen , H. Rosing , et al. 2022a. “Development and Validation of an HPLC‐MS/MS Method for the Quantification of the Anti‐Leishmanial Drug Miltefosine in Human Skin Tissue.” Journal of Pharmaceutical and Biomedical Analysis 207: 11402. 10.1016/J.JPBA.2021.114402. 34634528

[bmc70158-bib-0010] Roseboom, I. C. , B. Thijssen , H. Rosing , et al. 2022b. “Development and Validation of a High‐Performance Liquid Chromatography Tandem Mass Spectrometry Method for the Quantification of the Antiparasitic and Antifungal Drug Amphotericin B in Human Skin Tissue.” Journal of Chromatography B 1206: 123354. 10.1016/j.jchromb.2022.123354. 35810536

[bmc70158-bib-0011] Roseboom, I. C. , B. Thijssen , H. Rosing , et al. 2022c. “Development and Validation of an Ultra‐High Performance Liquid Chromatography Coupled to Tandem Mass Spectrometry Method for the Quantification of the Antileishmanial Drug Paromomycin in Human Skin Tissue.” Journal of Chromatography B 1211: 123494. 10.1016/j.jchromb.2022.123494. 36219923

[bmc70158-bib-0012] Schouten, W. M. , K. van Bocxlaer , H. Rosing , et al. 2025. “Development and Validation of Ultra‐Performance Liquid Chromatography Tandem Mass Spectrometry Methods for the Quantitative Analysis of the Antiparasitic Drug DNDI‐6148 in Human Plasma and Various Mouse Biomatrices.” Journal of Chromatography B 1250: 124377. 10.1016/j.jchromb.2024.124377. 39577310

[bmc70158-bib-0013] Stover, C. K. , P. Warrener , D. R. VanDevanter , et al. 2000. “A Small‐Molecule Nitroimidazopyran Drug Candidate for the Treatment of Tuberculosis.” Nature 405, no. 6789: 962–966. 10.1038/35016103. 10879539

[bmc70158-bib-0014] Thompson, A. M. , P. D. O'Connor , A. J. Marshall , et al. 2018. “Development of (6R)‐2‐Nitro‐6‐[4‐(Trifluoromethoxy)Phenoxy]‐6,7‐Dihydro‐5H‐Imidazo[2,1‐B][1,3]Oxazine (DNDI‐8219): A New Lead for Visceral Leishmaniasis.” Journal of Medicinal Chemistry 61, no. 6: 2329–2352. 10.1021/acs.jmedchem.7b01581. 29461823 PMC5867678

[bmc70158-bib-0015] Torreele, E. , B. Bourdin Trunz , D. Tweats , et al. 2010. “Fexinidazole—A New Oral Nitroimidazole Drug Candidate Entering Clinical Development for the Treatment of Sleeping Sickness.” PLoS Neglected Tropical Diseases 4, no. 12: e923. 10.1371/journal.pntd.0000923. 21200426 PMC3006138

[bmc70158-bib-0016] van Bocxlaer, K. , D. Caridha , C. Black , et al. 2019. “Novel Benzoxaborole, Nitroimidazole and Aminopyrazoles With Activity Against Experimental Cutaneous Leishmaniasis.” International Journal for Parasitology: Drugs and Drug Resistance 11: 129–138. 10.1016/j.ijpddr.2019.02.002. 30922847 PMC6904836

[bmc70158-bib-0017] van Bocxlaer, K. , K. N. McArthur , A. Harris , et al. 2021. “Film‐Forming Systems for the Delivery of DNDI‐0690 to Treat Cutaneous Leishmaniasis.” Pharmaceutics 13, no. 4: 516. 10.3390/pharmaceutics13040516. 33918099 PMC8069359

[bmc70158-bib-0018] van den Kerkhof, M. , D. Mabille , E. Chatelain , et al. 2018. “In Vitro and In Vivo Pharmacodynamics of Three Novel Antileishmanial Lead Series.” International Journal for Parasitology: Drugs and Drug Resistance 8, no. 1: 81–86. 10.1016/j.ijpddr.2018.01.006. 29425734 PMC6114106

[bmc70158-bib-0019] WHO . 2023a. “Global Leishmaniasis Surveillance, 2022: Assessing Trends Over the Past 10 Years.” Accessed 07‐02‐2025, from https://www.who.int/publications/i/item/who‐wer9840‐471‐487.

[bmc70158-bib-0020] WHO . 2023b. “Leishmaniasis. Key Facts.” Accessed 06‐02‐2025, from https://www.who.int/news‐room/fact‐sheets/detail/leishmaniasis.

[bmc70158-bib-0021] Wijnant, G. J. , S. L. Croft , R. de la Flor , et al. 2019. “Pharmacokinetics and Pharmacodynamics of the Nitroimidazole DNDI‐0690 in Mouse Models of Cutaneous Leishmaniasis.” Antimicrobial Agents and Chemotherapy 63, no. 9: e00829‐19. 10.1128/AAC.00829-19. 31262757 PMC6709472

