# Supplementary material for: Validated LC‐MS/MS Method for Quantifying the Antiparasitic Nitroimidazole DNDI‐0690 in Preclinical Target Site PK/PD Studies
Source: Biomed Chromatogr. 2025 Jun 26;39(8):e70158. doi: 10.1002/bmc.70158 (PMC12199288; doi:10.1002/bmc.70158)
Supplement: Supplementary file 1 — Table S1 Above: general mass spectrometric parameters. Below: Analyte specific mass spectrometric parameters for DNDI‐0690 and [d4]‐DNDI‐0690. Table S2 Calibration standard concentrations, back‐calculated accuracy (Bias, %) and precision (CV, %) of DNDI‐0690 (in ng/mL) for the three different sample preparation methods, analyzed in three consecutive analytical runs. [file BMC-39-e70158-s001.docx]

# SUPPLEMENTARY TABLES

**Table 1:** Above: general mass spectrometric parameters. Below: Analyte specific mass spectrometric parameters for DNDI-0690 and [d_4_]-DNDI-0690.

| **Mass-spectrometer** | |  |
| --- | --- | --- |
| **Run duration** | 4 min |  |
| **Polarity** | Positive |  |
| **Ionspray voltage** | 5500 V |  |
| **Nebulizer gas** | 55 psi |  |
| **Turbo gas/heater gas** | 40 psi |  |
| **Curtain gas** | 30 psi |  |
| **Collision gas** | 10 psi |  |
| **Temperature** | 500 °C |  |
|  |  |  |
|  | DNDI-0690 | [d_4_]-DNDI-0690 |
| **Transition (m/z)** | 371.0 🡪 189.0 | 375.0 →193.0 |
| **Collision energy (V)** | 121 | 121 |
| **Collision exit potential (V)** | 31 | 31 |
| **Declustering potential (V)** | 24 | 24 |
| **Entrance potential (V)** | 10 | 10 |
| **Dwell time (msec)** | 100 | 100 |

**Table 2:** Calibration standard concentrations, back-calculated accuracy (Bias, %) and precision (CV, %) of DNDI-0690 (in ng/mL) for the three different sample preparation methods, analyzed in three consecutive analytical runs.

| **Calibration standard** | **Human K_2_EDTA plasma** | | | **Enzymatic Digestion Buffer** | | | **Microdialysate** | | |
| --- | --- | --- | --- | --- | --- | --- | --- | --- | --- |
|  | **Conc. (ng/mL)** | **Bias (%)** | **CV**  **(%)** | **Conc. (ng/mL)** | **Bias (%)** | **CV**  **(%)** | **Conc. (ng/mL)** | **Bias (%)** | **CV**  **(%)** |
| **1 (LLOQ)** | 2.50 | -0.9 | 3.0 | 1.00 | -0.7 | 2.6 | 0.500 | 2.8 | 4.7 |
| **2** | 5.00 | 1.5 | 4.3 | 2.00 | 1.3 | 3.3 | 1.00 | -6.5 | 5.8 |
| **3** | 10.0 | -0.4 | 2.6 | 10.0 | 1.3 | 1.3 | 5.00 | -1.2 | 2.8 |
| **4** | 50.0 | 2.8 | 0.9 | 30.0 | 0.4 | 2.1 | 15.0 | 0.2 | 1.8 |
| **5** | 200 | 1.6 | 1.4 | 100 | 0.7 | 0.9 | 30.0 | 2.4 | 1.1 |
| **6** | 400 | -4.5 | 2.0 | 200 | -0.9 | 2.2 | 60.0 | 0.0 | 0.3 |
| **7** | 800 | -0.3 | 1.7 | 400 | -0.4 | 2.2 | 80.0 | -0.3 | 1.1 |
| **8 (ULOQ)** | 1,000 | -0.1 | 1.2 | 500 | -1.8 | 1.8 | 100 | 1.6 | 2.5 |

Abbreviations: Conc. = concentration; CV = coefficient of variation; LLOQ = Lower limit of quantification; ULOQ = Upper limit of quantification.
